# Role of successive round as a quality signal in equity crowdfunding: Novel evidence from the perspective of investors’ preferences

**DOI:** 10.1371/journal.pone.0297820

**Published:** 2024-03-07

**Authors:** Syed Muhammad Hamza Abid Wasti, Jaleel Ahmed, Mushtaq Hussain Khan

**Affiliations:** 1 Department of Management Sciences, Capital University of Science and Technology, Islamabad, Pakistan; 2 Department of Accounting and Finance, Capital University of Science and Technology, Islamabad, Pakistan; 3 Cardiff School of Management, Cardiff Metropolitan University, Cardiff, United Kingdom; University of Palermo, ITALY

## Abstract

Equity crowdfunding provides entrepreneurs and founders the opportunity to raise funds from a large number of potential investors, using quality signals to influence their investment decisions. Drawing from the lens of signaling theory and the elaboration likelihood model, this study explores the role of successive equity crowdfunding rounds as a quality signal in shaping investors’ preferences in crowdfunded firms and its influence on their investment decisions. Our findings reveal that successive equity crowdfunding rounds serve as quality signals, modeling investors’ preferences and thereby resulting in a high magnitude of success factors. The successive round is a strong quality signal that has a positive and significant impact on investors’ investment decisions in subsequent equity crowdfunding rounds. The increasing preferences of investors due to the successive round augments the magnitude of success factors and helps entrepreneurs in successfully achieving large funding targets, high overfunding, and attracting a large number of investors in subsequent equity crowdfunding campaigns, even with a low level of equity offering.

## Introduction

Crowdfunding helps entrepreneurs to transform their entrepreneurial competencies into entrepreneurial rewards [1]. Asymmetric information between potential investors and entrepreneurs, uncertainty of investment returns, local bias, and lack of collateral are the causes of financial constraints that lead to funding gaps for new ventures and entrepreneurs [2–8]. Besides, new ventures encounter difficulties in accessing external financing in their early stage of development due to the inclination of traditional investors (e.g., business angels, banks, and venture capitalists) to invest in less risky and more cost-effective opportunities within established firms. In such circumstances, entrepreneurs typically fund their ventures by utilising their own capital, gathering funds from family and friends, in order to prove their ideas and cover early startup costs [9]. Social networks are considered a solution to early-stage financing gaps, assuming that online social networks enable new ventures to access a novel source, namely crowdfunding [10–15]. Shiller [16] suggested that new ventures can resolve their financial issues through an innovative method of securitization named equity crowdfunding.

Interestingly, the extensive body of literature on various crowdfunding types comprehensively addresses both entrepreneurs and the crowd; however, when it comes to equity crowdfunding, the research predominantly centers on the entrepreneur [17]. Consequently, a major chunk of existing studies delves into the investigation of factors contributing to the success of crowdfunding campaigns. These studies examined how entrepreneurs can effectively persuade equity crowdfunders to invest during the campaign [18–23]. However, investors’ perspective is largely neglected in existing studies [17, 24].

Equity crowdfunding operates within the realm of the FinTech landscape [25], capitalizing on the presence of digital platforms that can assist entrepreneurs in overcoming financial constraints, especially during the early stages of business ventures [26]. In the context of digital platforms, Elaboration Likelihood Model (ELM) explains the impact of online information on investing decision of the funders in crowdfunding. This is a major theoretical model which is used in online behavior research [27–34]. Information about product quality and its specification is classified as the central route while online likes and comments are the peripheral route. These two routes of ELM have been studied in context of online purchasing [27, 35, 36]. In the specific case of crowdfunding, Bi et al. [37] were the first to introduce the ELM to the crowdfunding literature.

Our study examines the impact of quality signals (central route) and social network activities (peripheral route) of the ELM on overfunding in the context of equity crowdfunding, focusing on the investors’ perspective. Alternatively speaking, our study aims to empirically explore the role of successive equity crowdfunding campaign rounds as a quality signal in developing investors’ preferences for crowdfunded firms and its influence on investors’ investment decisions. In contrast to donation-based crowdfunding, equity crowdfunding involves higher levels of process complexity and risk [37]. Investors in this form of crowdfunding encounter a greater volume of information and have much deeper consideration [38, 39]. Equity crowdfunding platforms often provide due diligence services to assist online investors, and some investors request creators to provide a project finance roadmap [23]. These factors highlight the complex decision-making process within the context of equity-based crowdfunding, where investors exhibit varying perception paths and behavioral patterns. Previous research indicates that investors tend to behave like consumers in equity crowdfunding platforms, as the primary business model of equity crowdfunding involves “pre-selling” [38, 40, 41]. When contemplating whether to fund such “pre-selling” projects, investors show online behavior just like consumers buy goods [42]. Therefore, in the context of equity crowdfunding, the ELM can be employed to explore the factors influencing investment decisions concerning equity crowdfunding projects. Based on ELM, our study seeks to answer the following questions. First, do successive equity crowdfunding campaigns influence the number of investors offering investment in the project? Second, do successive equity crowdfunding campaigns help entrepreneurs raise the target amount of funding? Third, do successive equity crowdfunding campaigns impact the probability of achieving a high funding target? Finally, do successive equity crowdfunding campaigns influence overfunding?

Drawing on the lens of signaling theory proposed by Spence [43], existing studies have been examined the impact of campaign characteristics, online updates during the campaign, entrepreneurs’ information cascade and textual information on campaign success and overfunding [44–52]. Information asymmetry between entrepreneurs and investors is also a major concern in equity crowdfunding, similar to conventional venture capital financing [53–55]. Entrepreneurs usually possess greater knowledge about the quality of ventures than potential investors [56–58], and this information asymmetry becomes even more pronounced within the context of equity crowdfunding [25, 59]. Information asymmetries on equity crowdfunding platforms are relatively higher for equity crowdfunding ventures because most important things for early-stage investors is to gather information, monitor progress and provide inputs to ventures but cost of these activities are distance sensitive in equity crowdfunding [60]. It can be observed that, despite the presence of information asymmetries, entrepreneurs have managed to secure significant funding through crowdfunding platforms like Crowdcube, Seedrs, Kickstarter, and ASSOB. This indicates that investors have found ways to assess the information presented on these platforms and determine the quality of the listed ventures. Moreover, investors interpret certain information as indicators of quality and make investment decisions based on these signals, as not all ventures receive funding.

In this stream of research, existing studies also documented that past successful equity crowdfunding campaigns help firms in obtaining funds from venture capital [18–20, 44, 61–63]. It means that past successful equity crowdfunding campaigns can lower information asymmetry and works as quality signal in subsequent equity crowdfunding campaigns. There are also supporting findings from the study by Butticè et al. [64] that successful equity crowdfunding campaigns have a higher probability of attracting investments from venture capital firms than other sources of funding. It is also observed that entrepreneurs who sell a smaller fraction of their companies at listing and have more social capital have a higher probability for a successful campaign [47]. In the case of equity crowdfunding, Walthoff-Borm et al. [65] noted that firms listed on equity crowdfunding platforms serve as a last resort for those facing lower profitability, excessive debt levels, and a higher proportion of intangible assets, rendering them unable to secure funding from traditional financiers. Furthermore, investors’ expertise in picking an equity crowdfunding is explained more by their learned skills and financial literacy than the investors’ demographics [66]. Besides, early bird investors attract a large number of late investors [67], while crowdfunding performance impacts venture capital firms’ screening and investing decisions [55].

The contribution of our study is as follows. By combining the signaling theory and the ELM in the context of equity crowdfunding, our study complements the works of Bi et al. [37], Shneor and Munim [68], Xiang et al. [69], Wang and Yang [70], Kim and Petrick [71], Wang et al. [45], Jianghong et al. [72], and Wasti and Ahmed [73]. These studies have combined signaling theory and the Elaboration Likelihood Model to explain reward crowdfunding, emotional appeals in crowdfunding, brand promotion effects in crowdfunding, and tourism crowdfunding ventures. While our study contributes to this emerging debate by adding an investors’ perspective, examining the role of successive equity crowdfunding rounds as a quality signal in shaping investors’ preferences for crowdfunded firms and influencing their investment decisions. Based on an unbalanced panel of 829 startups out of 1081 equity crowdfunded campaigns, our panel regression results suggest that a successive equity crowdfunding campaign round serves as a strong quality signal, exerting a positive and significant impact on investors’ preferences. This enhances subsequent fundraising, thereby helping entrepreneurs achieve successful high fundraising campaigns.

The rest of the study is structured as follows. The next section provides the literature review and the development of hypotheses, followed by the data, methodology, and the discussion of empirical results. The final section provides the concluding remarks.

## Literature and hypothesis development

Crowdfunding has emerged as an alternative financing method stemming from microfinancing and the broader concept of crowdsourcing. Companies and ventures that fail to convince institutional and traditional financing agents for funding during their start-up stage can utilise the tool of crowdfunding to generate funds [42]. Investment based crowdfunding platforms offer new ventures and firms the opportunity to access a larger pool of funders compared to traditional financing platforms, such as banks and venture capitalists [74]. The factors that drive entrepreneurial ventures to successful fundraising have been of great interest to scholars, especially in the venture capital context [9, 44, 45]. It is also observed that the type of project is associated with the project’s success [75], and both social network size and project quality contribute to a successful campaign [42].

Equity crowdfunding predominantly serves as a means of fundraising, yet companies also pursue other objectives through their equity crowdfunding endeavors [22]. Beyond capital acquisition, these objectives encompass promotional and marketing initiatives, feedback collection, market testing, and the cultivation of relationships [76]. Moreover, the definition of successful campaigns extends beyond merely meeting financial targets; it includes campaigns that attract a substantial number of investors as well [22]. Notably, investor preferences diverge based on the crowdfunding model and smaller funding targets tend to capture investors’ interest in reward-based crowdfunding [77–79], in campaigns with high funding target in equity crowdfunding [22], product certification from stakeholders and provision of documents [23, 80], early funding from private networks and social media network [22]. These factors are positively associated with the number of investors in an equity crowdfunding campaign. Investors also tend to favour campaigns involving business-to-consumer products. Consequently, there exists a positive relationship between campaigns featuring business-to-consumer products and the number of investors, as noted by Li et al. [26]. Furthermore, costly signals, such as information about successful fundraising in the past, transmit a positive indication to potential investors regarding the quality of the venture. This, in turn, leads to a successful campaign, as highlighted by Di Pietro et al. [81]. Thus, this study conjectures that:

***H1*:** *Successive equity crowdfunding campaigns positively influence on number of investors offering investment in the project*.

Information asymmetry poses a significant hurdle to attracting investors to engage in equity crowdfunding campaigns on crowdfunding platforms. Xiang et al. [69] concluded that information asymmetry within crowdfunding can influence the investment choices made by crowd investors. In order to mitigate this asymmetry, entrepreneurs use signaling mechanism, wherein various indicators of quality are communicated. An additional dimension of quality signaling is the contribution made by the founders, which has an impact on both the uncertainty surrounding the firm and the investment decisions of potential backers [71]. Likewise, Blaseg et al. [25] suggested that the proportion of equity that entrepreneurs hold within the context of a fundraising campaign emerges as a potent indicator of venture quality. This phenomenon stems from the fact that entrepreneurs incur costs to retain their equity stake. As a result, they are inclined to maintain a substantial equity interest in the project only if they foresee robust future cash flows from the company. They further suggest that a substantial equity stake in the firm serves to align the interests of the founders and the funding contributors, enhancing cohesion between these stakeholders.

A higher level of equity ownership can amplify the likelihood of achieving fundraising success, but it concurrently escalates the financial burden on entrepreneurs due to the necessity of securing a significant number of resources to effectively communicate the quality of the venture to potential investors. Notably, signals that come at a higher cost, such as demonstrations of previous successful crowdfunding campaigns and fundraising achievements, hold greater value for prospective investors compared to signals that incur no cost [81]. Consequently, in subsequent equity crowdfunding campaigns, the accomplishments of prior successful crowdfunding efforts can function as strong and costly signals in achieving high funding targets, even with a lower level of equity interest in the venture during subsequent fundraising campaigns. Therefore, this study assumes that:

***H2*:** *Successive equity crowdfunding campaigns help entrepreneurs to raise the target amount of funding even with lower level of equity offering in the project*.

In an important study, Mazzocchini and Lucarelli [82] conducted a systematic literature review on factors related to success or failure in equity crowdfunding. The factors influencing success or failure during crowdfunding projects include web presence, updates, founder teams, firm age, industry sector, number of founders or CEOs, founder gender, entrepreneurs’ financial commitment, informational asymmetries among entrepreneurs and investors, and the number of comments on social media, among others [77, 83–85]. Likewise, larger word counts in introduction and larger video counts are associated with reward-based crowdfunding as these make the funder feel the project of higher quality [86]. Moreover, online reviews and likes make the funder feel the project of having good electronic word of mouth [37].

Equity crowdfunding is a significant financial innovation that enables new ventures and projects to raise the required capital [16], and its inevitability is expected to grow [25, 26]. Compared to traditional financiers, where interactions are limited to a few institutions or experienced individuals, an equity crowdfunding campaign, relying on the internet, can easily and swiftly reach a large crowd of potential investors [87]. Therefore, there is significantly lower cost and risk for each investor in the project compared to traditional financing models [88]. Equity crowdfunding has successfully established a niche in the market for startups and early-stage investments [89].

The signaling theory can also be applied in the context of equity crowdfunding, as evidenced by studies such as those by [23, 54, 67, 90]. This application mirrors its use in conventional financing, serving as a framework to examine investors’ decision-making processes and their selection of investment opportunities. Drover et al. [55] concluded that the performance of crowdfunding exerts a positive influence on the screening and investment choices made by venture capital firms. Further supporting this, Butticè et al. [64] documented that a successful equity crowdfunding campaign can mitigate information asymmetry while concurrently serving as a favourable indicator for investors in subsequent fundraising rounds. This assertion is underpinned by a positive correlation between companies that achieve success in their equity crowdfunding campaigns and the likelihood of securing funding from venture capital firms [61].

There are also opposing perspectives suggesting that crowd investors’ investment decisions are influenced by factors such as their experiences, personal preferences, geographical proximity, and peer influence, as highlighted by Di Pietro et al. [81], Shafi [91], and Wallmeroth [92]. Additionally, some studies explore the notion that a considerable number of crowd investors do not engage in an extensive evaluation process prior to making investment decisions in equity crowdfunding campaigns, even when it comes to analysing the forecasted financial returns of companies. This perspective is explored in works by Zinecker et al. [93] and Cumming et al. [94]. However, in a more recent study, Wasti and Ahmed [73] noted that investors give more weightage to quality signals than social network activities while taking investment decision in equity crowdfunding. Thus, this study conjectures that:

***H3*:** *Successive equity crowdfunding campaigns impact the probability of achieving a high funding target*.

Empirical evidence also suggests that certain campaigns experience higher degrees of overfunding compared to others [95]. This phenomenon of overfunding holds significant relevance for entrepreneurs as it allows them to secure additional resources beyond their initial funding goals, aiding in covering startup cost. Notably, overfunding serves as a valuable mechanism for entrepreneurs to accumulate supplementary funds for their projects. Consequently, entrepreneurs not only accept overfunding but also leverage it as a quality signal to draw in a larger pool of investors. Extant literature also reveals that quality signals like provision of documents [42], provision of financial projection in equity crowdfunding [96], length of the project description [6], provision of video [62] positively influences funding success in crowdfunding, and magnitude of these quality signals as success factors increase the overfunding of a campaign [95]. A study by Hornuf et al. [97] investigated the connection between a firm’s achievement of a successful equity crowdfunding campaign and its higher likelihood of securing funds from venture capital firms Their findings imply that a successful equity crowdfunding campaign can serve as a quality signal for investors in subsequent fundraising rounds, potentially contributing to the phenomenon of overfunding in subsequent equity crowdfunding campaign.

We follow existing studies to theoretically establish the link between ELM and equity crowdfunding campaigns in the context of overfunding. The innovation diffusion theory [98] discusses how persuasion and influence processes shape human perceptions and behavior. According to this theory, external information introduces individuals to new possibilities, prompts a reexamination of their existing beliefs and attitudes, and can potentially alter their behaviors. Building upon the framework of the innovation diffusion theory, the ELM categorises persuasion mechanisms or routes into central and peripheral types, depending on the type of information processed by the user [99]. For instance, task-relevant information and social network activities are considered as central and peripheral types, respectively. In the specific case of equity crowdfunding, external information plays a crucial role in shaping the herd behavior of investors due to information asymmetry [100]. In crowdfunding, founders often possess more information about the underlying quality of the project than potential investors, resulting in informational disadvantages for investors regarding the founder’s credibility to deliver the promised product or service [23, 42]. Intriguingly, Li et al. [101] observed that equity crowdfunding campaigns result in overfunding due to herding behavior among investors, driven by persuasion. They also suggested that the formation of an initial herd acts as persuasion, probing investment interest in a particular campaign. This initial momentum then triggers a bandwagon effect, enticing funders to join the campaign and ultimately leading to overfunding. Hence, this study assumes that:

***H4*:** *Successive equity crowdfunding campaigns positively influence overfunding*.

## Data and research methodology

### Construction of the sample

Data regarding variables of interest are obtained from the world’s largest equity crowdfunding platform, Crowdcube [data in S1 Appendix]. Crowdcube is the first equity crowdfunding platform established in the United Kingdom. Crowdcube equity crowdfunding platform offers a unique database for empirical research. It is authorised and regulated by the Financial Conduct Authority under the Bank of England. Crowdcube reports that this platform has raised £1 billion for around 1,120 successfully funded projects. Moreover, there are 900,000 registered members, and the average investment amount for a project is £692,000. Data on campaign characteristics are collected from the campaign page on Crowdcube, and directors’ information is sourced from Companies House. To explore the impact of later rounds on investors’ preferences, the sample comprises an unbalanced panel of 829 startups out of 1081 equity crowdfunded campaigns conducted between July 2011 and December 2022, after excluding 43 companies due to incomplete data. Table 1 provides the definitions of variables used in this study.

**Table 1:** Description of variables.

Variable	Definition
Successive Round	This represents the number of successfully funded equity crowdfunding campaigns on Crowdcube. It is measured by assigning numbers to the first, second, third, fourth, fifth, and sixth successive rounds in chronological order based on date.
Target	It is an amount that the entrepreneur wants to collect in equity crowdfunding campaign for a project. It is measured by taking the natural logarithm of total amount of target.
Overfunding	This represents the percentage of the raised amount in a successful campaign compared to the target amount.
Equity	This refers to the amount contributed by the campaign promoters to a project. It is expressed as a percentage of the target amount offered by entrepreneurs.
Investors	The total number of investors who have invested in a specific firm. It is calculated as the natural logarithm of the total number of investors who have participated in an equity crowdfunding campaign.
Largest Investment	This refers to the largest investment made by a single investor in a firm. It is calculated as the natural logarithm of the amount invested by a single investor or venture capital.
Financial Forecast	This is the financial forecast provided by entrepreneurs in some equity crowdfunding campaigns. It takes value “1” if the financial documents provide forecasted revenue and earnings before interest, taxes, depreciation, and amortization (EBITDA), and “0” otherwise.
Directors	The total number of directors running an equity crowdfunded business. It is calculated based on the total number of directors that a firm has during the equity crowdfunding campaign.
Foreign Directors	Foreign directors are defined as directors who hold a nationality other than British nationality. It is calculated based on the total number of foreign directors in a firm during the equity crowdfunding campaign.
CF Experience	This refers to the prior involvement or experience of directors in equity crowdfunding on a crowdfunding platform. It takes value “1” when there are directors with crowdfunding experience, and “0” otherwise.
Social Forums	It is calculated based on the total number of online social media accounts that a company is utilising during the equity crowdfunding campaign.

Table 2 presents the summary statistics and correlation matrix of all variables. As indicated in Table 2, correlations between dependent and independent variables are significant and positive. Successive rounds show significant positive correlation with almost all dependent variables.

**Table 2:** Descriptive statistics and correlation matrix.

Variables	Mean	SD	1	2	3	4	5	6	7	8	9	10	11
Successive Round	1.290	0.670	1										
Overfunding	163.041	87.670	0.571***	1									
Target	12.380	0.985	0.332***	0.289***	1								
Equity	14.452	7.470	-0.371***	-0.206***	-0.116***	1							
Investors	5.725	1.096	0.338***	0.427***	0.841***	-0.139***	1						
Largest Investment	11.097	1.236	0.431***	0.386***	0.438***	-0.153***	0.327***	1					
Financial Forecast	0.533	0.505	0.127***	0.268***	0.218***	0.028	0.219***	0.193***	1				
Directors	2.946	1.805	0.138***	0.184***	0.311***	-0.124***	0.273***	0.268***	0.271***	1			
Foreign Directors	0.572	0.997	0.096***	0.143***	0.212***	-0.116***	0.244***	0.202***	0.166***	0.417***	1		
CF Experience	0.271	0.445	0.407***	0.341***	0.411***	-0.171***	0.357***	0.401***	0.296***	0.423***	0.253***	1	
Social Forums	2.944	1.092	0.073**	0.174***	0.156***	-0.091***	0.176***	0.139***	0.219***	0.168***	0.118***	0.2	1

### Methodology

In order to test our hypotheses, the regression-based model is employed. The logarithmic transformation of target amount, number of investors, and largest investment is used because it reduces the skewness of the variables and enhances the models’ fitness [22]. The following econometric models elucidate the linear relationship between dependent and independent variables.

Eq 1 examines the impact of successive equity crowdfunding rounds on number of investors.


$${Investors}_{i,t}=\beta_{o}+\beta_{1}{Successive\;Round}_{i,t}+\beta_{2}{Directors}_{i,t}+\beta_{3}{Financial\;Forecast}_{i,t}+\beta_{4}{Largest\;Investment}_{i,t}+\beta_{5}{Social\;Forums}_{i,t}+\mu_{i,t}$$ (1)

Eq 2 investigates the impact of successive equity crowdfunding rounds on equity offering.


$${Equity\;offering}_{i,t}=\beta_{o}+\beta_{1}{Successive\;Round}_{i,t}+\beta_{2}{Target}_{i,t}+\beta_{3}{Directors}_{i,t}+\beta_{4}{Foreign\;Directors}_{i,t}+\beta_{5}{Social\;Forums}_{i,t}+\mu_{i,t}$$ (2)

Eq 3 explores the impact of successive equity crowdfunding rounds on target.


$${Target}_{i,t}=\beta_{o}+\beta_{1}{Successive\;Round}_{i,t}+\beta_{2}{Financial\;Forecast}_{i,t}+\beta_{3}{Directors}_{i,t}+\beta_{4}{CF\;Experience}_{i,t}+\beta_{5}{Social\;Forums}_{i,t}+\mu_{i,t}$$ (3)

Eq 4 tests the impact of successive equity crowdfunding round on overfunding.


$${Overfunding}_{i,t}=\beta_{o}+\beta_{1}{Successive\;Round}_{i,t}+\beta_{2}{Target}_{i,t}+\beta_{3}{Directors}_{i,t}+\beta_{4}{Largest\;Investment}_{i,t}+\beta_{5}{Investors}_{i,t}+\mu_{i,t}$$ (4)

Here in Eqs 1–4, successive round is the explanatory variable, while the others are control variables. The control variables are identified based on the equity crowdfunding literature. Controlling for directors is crucial, as suggested by [20, 47], directors play a pivotal role in the success of equity crowdfunding, attributable to the quality of human capital. Furthermore, the signaling value in financial forecasts becomes particularly significant, as the impact of quality signal generated through financial forecasts is more pronounced in equity crowdfunding, especially when information asymmetries are high [23, 102]. Controlling for large investments is imperative, as indicated by Vulkan et al. [103] and Décarre and Wetterhag [104], who suggested that a substantial investment may signal the involvement of venture capital or business angels as professional investors in the context of equity crowdfunding. Finally, social forums also play an important role in equity crowdfunding, as utilising a maximum number of popular social forums allows founders to reach a broader chunk of potential investors. In this context, Wasti et al. [73] documented that likes and shares on social forums contribute to funders perceiving the project as having positive electronic word of mouth, influencing investors’ investment decisions.

## Results and discussion

### Impact of successive round on investors’ preferences

In order to model the investors’ preferences and successive round, this study proxied the investors’ preferences through subsequent campaign success with high targets, high fund raising, high overfunding, low equity requirement, large number of investors, and large investments from professional investors. Regression based approach is used to explore the relationship between successive rounds and different measures of investors’ preferences along with the control variables.

Table 3 reports the results regarding the impact of successive crowdfunding rounds on number of investors in equity crowdfunding.

**Table 3:** The impact of successive crowdfunding rounds on number of investors in equity crowdfunding.

Variables	Coefficient	Standard Error	t-value	p-value
Successive Round	0.379	0.049	7.783	0.000
Directors	0.099	0.018	5.518	0.000
Financial Forecast	0.623	0.053	11.726	0.000
Largest Investment	0.0000005	0.0000001	4.287	0.000
Social Forums	0.279	0.025	11.072	0.000
Const.	3.709	0.100	37.065	0.000
Observations	1081			
Adj. R—square	0.438			
F–static	169.471			
Probability (F–static)	0.000			

The findings in Table 3 show that 43.8 percent of the variance in the number of investors in equity crowdfunding campaigns is explained by the explanatory variables. The coefficient of successive round is positive and significant (p<0.01) in relation to number of investors. Thus, our first hypothesis is accepted which conjectures that successive equity crowdfunding campaign positively influence on number of investors offering investment in the project. Moreover, the coefficients of all control variables are positive and significant in relation to number of investors in equity crowdfunding. These findings suggest that with the increase in number of successive rounds, number of investors also increases. This is because a successive round after a successful equity crowdfunding campaign shows past achievement of successful fund raising by a firm. Studies suggest that past achievements work as costly signals for potential investors [81]. Thus, subsequent rounds also work as costly signals which have more attraction for investors than costless signals. Successive rounds are positively and significantly associated with the largest investment in equity crowdfunding campaigns. Largest investment generally shows the presence of professional investors in equity crowdfunding. Subsequent equity crowdfunding campaigns may have greater attraction not only for small investors but also for business angels and venture capitalists to pledge their large investments in equity crowdfunding campaigns [97]. Similar findings are also reported by Butticè et al. [64] that successful equity crowdfunding campaigns have more probability of attracting investments from venture capital firms than other sources of funding.

Table 4 reports the results regarding the impact of successive crowdfunding rounds on equity offering in equity crowdfunding.

**Table 4:** The impact of successive crowdfunding rounds on equity offering in equity crowdfunding.

Variables	Coefficient	Standard Error	t-value	p-value
Successive Round	-4.101	0.324	-12.640	0.000
Target	0.00000043	0.00000045	0.940	0.348
Directors	-0.213	0.131	-1.623	0.105
Foreign Directors	-0.470	0.199	-2.361	0.018
Social Forums	-0.382	0.199	-1.917	0.056
Const.	21.589	0.750	28.772	0.000
Observations	1081			
Adj. R—square	0.145			
F–static	37.891			
Probability (F–static)	0.000			

The findings in Table 4 show that 14.5 percent of the variance in equity offered in the equity crowdfunding campaign is explained by the explanatory variables. The coefficient of successive round is negative and significant (p<0.01) in relation to the equity offered. The coefficients of foreign directors and social forums are also negative and significant (p<0.05) in relation to equity offered in equity crowdfunding. Hence, our second hypothesis is accepted which assumes that successive equity crowdfunding campaigns enable entrepreneurs to raise the target amount even with lower level of equity offering in the project. The significant and negative impact of successive round on equity offered implying that with the increase in number of successive round, there is decrease in equity offered by entrepreneur in subsequent rounds. Although existing studies have documented that the equity offered is positively associated with campaign success because equity contribution by founders is a sign of quality that impacts the uncertainty about the firm and also influences investors’ investment decisions [73, 81], our results suggest that successive round in equity crowdfunding is a stronger quality signal than an equity signal. This helps entrepreneurs to attract investors with low equity contributions in subsequent crowdfunding campaigns. Thus, a subsequent equity crowdfunding campaign may help to achieve high funding targets even with lower level of equity interest in the venture in subsequent fund-raising campaign. These results are in line with the findings of studies by [95, 97].

Table 5 presents the results regarding successive crowdfunding rounds and high target amount in equity crowdfunding campaigns.

**Table 5:** The impact of successive crowdfunding rounds on high funding target.

Variables	Coefficient	Standard Error	t-value	p-value
Successive Round	0.075	0.034	2.196	0.008
Financial Forecast	0.351	0.044	7.938	0.000
Directors	0.115	0.013	8.916	0.000
CF Experience	0.979	0.058	16.979	0.000
Social Forums	0.130	0.020	6.525	0.000
Const.	11.112	0.077	143.583	0.000
Observations	1081			
Adj. R—square	0.518			
F–static	232.792			
Probability (F–static)	0.000			

The findings show that 51.8 percent of the variance in the target amount in equity crowdfunding is explained by the explanatory variables. The results further reveal that the coefficient of successive round is significant and positive at 99% significance level (Beta = 0.074679) implying that as the increase in the number of subsequent rounds, there is an increase in the successfully collected target amount within an equity crowdfunding campaign. Hence, our third hypothesis is accepted which conjectures that successive equity crowdfunding campaign increases the probability to achieve high funding target. Alternatively speaking, our findings suggest that a subsequent equity crowdfunding campaign can work as a quality signal and develop trustworthiness between entrepreneur and investors. Our findings are consistent with the existing studies suggesting that a subsequent funding round is a good predictor of firm survival [97] that can signal to investors about the potential of a firm’s future success. Thus, investors make investment decisions in subsequent equity crowdfunding campaigns that may results in achieving high funding targets and may result in high fundraising against targeted amount in successive rounds [61, 64, 81]. In the case of control variables, as expected, our results suggest a significant and positive impact on the target amount.

Table 6 reports the results regarding the impact of successive crowdfunding rounds on overfunding in the equity crowdfunding.

**Table 6:** The impact of successive crowdfunding rounds on overfunding in equity crowdfunding.

Variables	Coefficient	Standard Error	t-value	p-value
Successive Round	56.883	3.383	16.814	0.000
Target	-0.0000483	0.000005	-9.587	0.000
Directors	2.535	1.174	2.158	0.031
Largest Investment	0.000049	0.000007	6.846	0.000
Investors	0.034	0.003	13.216	0.000
Const.	73.479	5.417	13.564	0.000
Observations	1081			
Adj. R—square	0.444			
F–static	173.788			
Probability (F–static)	0.000			

The findings in Table 6 show that 44.4 percent of the variance in overfunding in equity crowdfunding is explained by explanatory variables. The coefficient of successive round is positive and significant (p<0.01) in relation to the overfunding. Hence, our fourth hypothesis is accepted which assumes that successive equity crowdfunding campaigns positively influence overfunding. Moreover, the coefficients of all control variables are also positive and significant in relation to overfunding in equity crowdfunding. Our findings are in line with the prior studies suggesting that subsequent funding rounds attract professional investors [97] whose participation in equity crowdfunding campaigns increase overfunding in equity crowdfunding campaign [95].

## Conclusion

Utilising the perspectives of signaling theory and the elaboration likelihood model, this study investigates whether successive rounds of equity crowdfunding function as signals of quality. It delves into how these quality signals contribute to develop trust among investors in crowdfunded enterprises and examines their impact on the investment choices made by these investors. Our findings suggest that successive rounds are strong quality signals that have a positive and significant impact on investors’ preferences, which, in turn, enhances subsequent fundraising. These findings are important because an increase in investors’ preferences due to successive rounds increases the magnitude of success factors and assists entrepreneurs in achieving successful high fundraising campaigns. Besides, successive rounds help firms meet high funding targets and achieve high overfunding, which is the most important desire of entrepreneurs. Intriguingly, a high level of equity is associated with campaign success, but successive rounds help entrepreneurs achieve high fundraising even with a low level of equity offering. Successive rounds not only attract crowd investors but also professional investors like venture capitalists and angel investors. As a result, an increase in the number of successive rounds leads to larger investments. The findings of this study provide important insights for entrepreneurs and investors when making rational decisions.

Previous studies suggest that small targets are associated with campaign success, while large targets can fulfill the funding requirements of a business but come with a risk of an unsuccessful campaign. This study assists entrepreneurs in devising a strategy regarding campaign success and larger funding targets. In this regard, entrepreneurs can set small funding targets in their initial campaign to ensure campaign success. Subsequently, they can leverage the success of that previous campaign as a quality signal to attain larger funding targets in subsequent crowdfunding rounds. Similarly, this study is also beneficial for investors when selecting investment opportunities, as subsequent rounds serve as good predictors of post-campaign firm success. This information aids investors in including successive rounds in their evaluation of investment opportunities, potentially assisting them in mitigating investment risks in equity crowdfunding.

This research can be extended in the following directions. Firstly, while this study examines successive rounds within the context of equity crowdfunding, future research could enhance our findings by investigating the applicability of successive rounds as quality signals in other forms of crowdfunding, such as reward-based crowdfunding and peer-to-peer lending. Secondly, this study uses data from a single crowdfunding platform. However, future researchers could broaden the scope by incorporating diverse equity crowdfunding platforms from various countries, allowing for the generalisation of our findings.

## Supporting information

S1 Appendix (XLSX)
